# Preparation and Characterization of Novel Sulfoaluminate-Cement-Based Nonautoclaved Aerated Concrete

**DOI:** 10.3390/ma17040836

**Published:** 2024-02-09

**Authors:** Feifei Peng, Chang Chen, Shaowu Jiu, Qiang Song, Yanxin Chen

**Affiliations:** College of Materials Science and Engineering, Xi’an University of Architecture & Technology, Xi’an 710055, China; pengfeifei@xauat.edu.cn (F.P.); jiushaowu@xauat.edu.cn (S.J.); songqiang123@xauat.edu.cn (Q.S.)

**Keywords:** nonautoclaved aerated concrete, mechanical properties, pore structure analysis, microstructural analysis

## Abstract

The production of autoclaved aerated concrete via the autoclaving process incurs substantial energy consumption, posing a challenge to sustainable economic development. Herein, a novel nonautoclaved aerated concrete (NAAC) was prepared using sulfoaluminate cement as the primary raw material and aluminum powder as the aerating agent. The physicomechanical characteristics and pore structures of the sulfoaluminate-cement-based (SAC) NAAC (SAC-NAAC) were examined through X-ray diffraction, thermogravimetry, and scanning electron microscopy. The findings revealed that the optimal mechanical attributes of the SAC-NAAC were achieved at a water–cement ratio of 0.55, with a specific content ratio of polycarboxylate superplasticizer–borax–calcium stearate–sodium hydroxide at 0.24%:0.32%:0.36%:2.90%, along with 0.40% aluminum powder. The SAC-NAAC samples, with a bulk density range of 600–750 g/m^3^, exhibited a compressive strength of 3.55–4.16 MPa, porosity of 45.9–63.5%, and water absorption rate of 60.2–74.4%. The weight loss in the SAC-NAAC with different aluminum powder contents ranged between 15.23% and 16.83%. The prismatic ettringite (AFt) crystals served as the main source of strength for the SAC-NAAC, and AH_3_ was attached to the AFt surfaces in a microcrystalline gel phase, thereby further enhancing the strength of the SAC-NAAC. Thus, the lightweight, high-strength SAC-NAAC has great potential as a nonautoclaved aerated concrete.

## 1. Introduction

The emphasis on building energy efficiency is increasing with the rising energy consumption in buildings. Additionally, assembled building structures are garnering attention owing to their ability to diminish dust and noise pollution during construction, conserve materials in transportation, and expedite the construction timeline. As a preassembled building material, autoclaved aerated concrete (AAC) primarily comprises calcareous materials, siliceous materials, aerating agents, and gypsum. Its fabrication involves batching, mixing, pouring, preconditioning, cutting, autoclaving, and curing [1,2]. Liu et al. [3] utilized ordinary silicate cement as the cementitious material and aluminum powder as the foaming agent to produce AAC. However, this process involved a precuring stage at 60 °C and an 18 h autoclave treatment at 2 MPa and 210 °C.

Similarly, Chen et al. [4] investigated the effects of the raw material proportions, foaming agent admixtures, and curing conditions in AAC production, which involved precuring at 23 ± 2 °C for 24 h, followed by a 16 h autoclave phase at 160–208 °C and a steam pressure of 1.2 MPa. Their study highlights the requisite of high-temperature and high-pressure steam curing in traditional AAC fabrication, which extends the production cycles, increases facility costs, and raises safety concerns.

Currently, research on nonautoclaved aerated concrete (NAAC) has garnered widespread interest. However, challenges such as its low strength and poor durability persist [5]. Using ordinary silicate cement, Jiang [6] experienced foam collapse in NAAC due to a mismatch between the setting and aerating periods. To address the strength limitations of NAAC, various studies have implemented short-term high-temperature curing. For instance, Aurélie et al. found that increasing the curing temperature could effectively improve the mechanical properties of nonautoclaved aerated concrete (NAAC) [7]. Xia et al. [8] enhanced the mechanical performance of NAAC by raising the curing temperature to 90 °C. Esmaily et al. [9] achieved high-performance NAAC through steam curing, with temperatures ranging from 70–87 °C. Similarly, Yang et al. [10] increased the steam temperature to 90 °C and fabricated NAAC that satisfied the standard requirements for compressive strength and frost resistance. However, preparing NAAC at ambient temperature and pressure without requiring high-temperature autoclave-curing equipment offers a simplified production process while reducing costs and energy consumption. Therefore, mastering the preparation of NAAC with stable properties at 20 °C represents a notable challenge in the contemporary construction industry [5].

As a principal cementitious material, fast-hardening cement facilitates the preparation of NAAC, enabling adjustments in the setting time and enhancing the compressive strength [11]. Sulfoaluminate cement (SAC) is a commonly used fast-hardening cement that exhibits benefits such as rapid setting and hardening, a high early strength, and minimal shrinkage, thereby imparting a high early strength to NAAC [12].

Li et al. [13] successfully developed high-strength NAAC using SAC as the cementitious material and sodium bicarbonate as the foaming agent (introducing CO_2_ gas into the SAC slurry). Nonetheless, the vigorous reaction of NaHCO_3_ creates difficulty in controlling the aerating rate, which renders it less suitable for large-scale production. Although aluminum powder is the most widely utilized aerating agent in practical applications owing to its more stable aerating process [14], it has not been extensively studied in the context of SAC-based NAAC (SAC-NAAC). Therefore, this study aimed to synchronize the setting time of SAC and the aerating time of aluminum powder by adding admixtures, especially NaOH. Ultimately, SAC-NAAC was successfully prepared using aluminum powder as the foaming agent at normal temperatures and under normal pressure conditions.

In this study, sulfoaluminate cement and aluminum powder were used as the cementitious material and foaming agent, respectively, to prepare the SAC-NAAC, considering the synergistic effects of sodium hydroxide, polycarboxylate superplasticizer, borax, and calcium stearate. The physical and mechanical properties of the SAC-NAAC were investigated at different mix ratios. Additionally, the macroscopic pore structure of the SAC-NAAC was analyzed using a digital camera, with fractal dimension verification, while its phase composition was examined through X-ray diffraction (XRD). The microscopic properties of the SAC-NAAC were further explored through scanning electron microscopy (SEM).

## 2. Materials and Methods

### 2.1. Materials

Sulfoaluminate cement (42.5 grade) was sourced from Tangshan Polar Bear Building Materials Co. (Tangshan, China). The main constituents are shown in Table 1, and it had a specific surface area > 360 m^2^/kg. Industrial-grade aluminum powder (fineness of 300 mesh) was purchased from Shaanxi Ningyuan Building Materials Co., Ltd. (Xixian New Area, China). Polycarboxylate superplasticizer (AR grade, PC8300, creamy yellow powder, water reduction rate of 30%) was procured from Jiangsu Zhaojia Building Materials Science and Technology Co., Ltd. (Xuzhou, China). Borax (AR grade, white crystalline powder, easily soluble in water, molecular formula Na_2_B_4_O_7_·10H_2_O, content ≥ 99.5%.) was obtained from Shaanxi Hailian Chemical Glass Instrument and Chemical Co. (Xian, China). Additionally, sodium hydroxide (AR grade, white crystalline particles, easily soluble in water, molecular formula NaOH, content ≥ 96.0%) and calcium stearate (AR grade, white powder, insoluble in water, CaO content 8.1–9.8%, specific surface area of 4.73–8.03 m^2^/g) were purchased from Tianjin Damao Chemical Reagent Co. (Tianjin, China).

### 2.2. Preparation of Sulfoaluminate-Cement-Based Nonautoclaved Aerated Concrete

**Figure 1:**
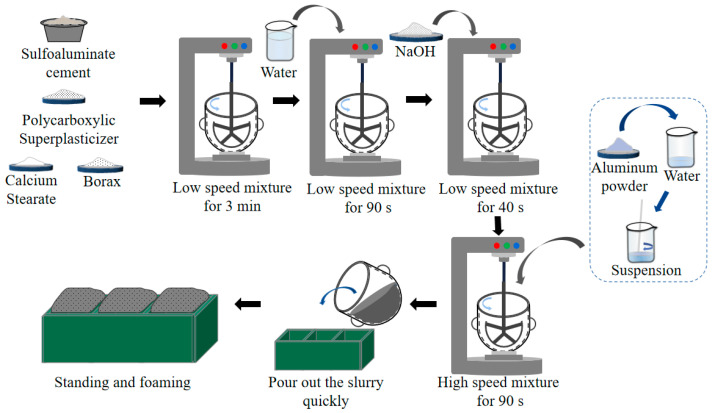
Preparation process of sulfoaluminate-cement-based nonautoclaved aerated concrete (SAC-NAAC).

The preparation methodology of the SAC-NAAC is depicted in Figure 1. Initially, sulfoaluminate cement, polycarboxylate superplasticizer, borax, and calcium stearate were mixed at a rotation speed of 140 ± 5 r/min for 3 min by following the prescribed mixture ratios. Subsequently, water was introduced into this powder blend and stirred at the same rotational speed for 90 s. Thereafter, sodium hydroxide was incorporated into the paste by mixing at 140 ± 5 r/min for 40 s. Finally, an aluminum powder suspension made by mixing aluminum powder and 30 g of water was rapidly added, and the resultant mixture was stirred at a higher speed of 285 ± 10 r/min for 90 s before being transformed into a mold (dimensions: 100 mm × 100 mm × 100 mm). After a precuring phase at 20 ± 2 °C for 6 h, the expanded portion was removed using a saw. Finally, the SAC-NAAC samples were demolded after 24 h.

### 2.3. Characterizations

In this study, the compressive strength, dry density, and water absorption were all determined using the GB/T 11969-2020 “Test methods of autoclaved aerated concrete”. In addition, since samples with different dry densities could not be compared in terms of their compressive strengths, the specific strength (ratio of the compressive strength to the dry density) was used as a criterion for selecting the optimal content. All data were taken from the arithmetic mean of a set of three samples.

Three samples of each SAC-NAAC group were placed in an electric blast-drying oven, initially set at 60 ± 5 °C for 24 h, followed by a temperature increase to 80 ± 5 °C for another 24 h, and they were ultimately maintained at 105 ± 5 °C until a constant quality was achieved. The dry density was determined as the arithmetic average of the dry densities of the three samples. Subsequently, the dried specimens were immersed in water, initially at one-third of the specimen’s height, for 24 h and then raised to two-thirds for another 24 h, and they were finally submerged entirely with an additional 30 mm of water for 24 h.

The mass of each specimen was recorded after 1, 6, 12, 24, 48, and 72 h of immersion to calculate the water absorption. Compressive strength testing was conducted using a Compression Testing Machine (TYE-2000C, Wuxi Building Materials Machinery Co., Ltd., Wuxi, China), wherein the applied pressure was directed perpendicular to the gas emission direction of the specimen with an accelerated load of (2.0 ± 0.5) kN/s.

For the pore size and distribution analysis of the SAC-NAAC samples, the samples were cut by means of a diamond saw, and all the pores in the cross-section of each sample were counted. The ImageJ2 software was employed to binarize the cross-section of the SAC-NAAC samples. The fractal dimension was used to assess the uniformity of the pore structure distribution. The composition of the SAC-NAAC samples was examined using an X-ray diffractometer (D8 Advance, Bruker AG, Bremen, Germany). The experiments were performed with a voltage of 40 kV, a current of 30 mA, and a scanning speed of 10°/min over a scanning range of 10° to 80°. A thermogravimetric analyzer (TG) that was a Netzsch TG 209 F3 Tarsus model from Germany was used to analyze the samples for the loss of weight substances. The test gas was nitrogen, the test temperature range was 30–500 °C, and the heating rate was 5 °C/min. A digital camera (DSC-T900, NETZSCH Instruments Manufacturing Co., Selb, Germany) captured images of the macroscopic pore structure, and the microscale pore structure of the SAC-NAAC samples was examined using a scanning electron microscope (EVO 10/LS 10, Carl Zeiss AG, Guina, Germany). The samples were sprayed with gold before shooting, fixed in the center of the carrier stage with conductive adhesive, and then put into the scanning electron microscope to observe the morphology of the sample cross-section. The accelerating voltage was 3 kV for shooting, 15 kV for spectral mapping, and the detector was a SE2 secondary electron detector.

## 3. Results

### 3.1. Effect of Water–Cement Ratio on the Properties of Sulfoaluminate-Cement-Based Nonautoclaved Aerated Concrete

**Figure 2:**
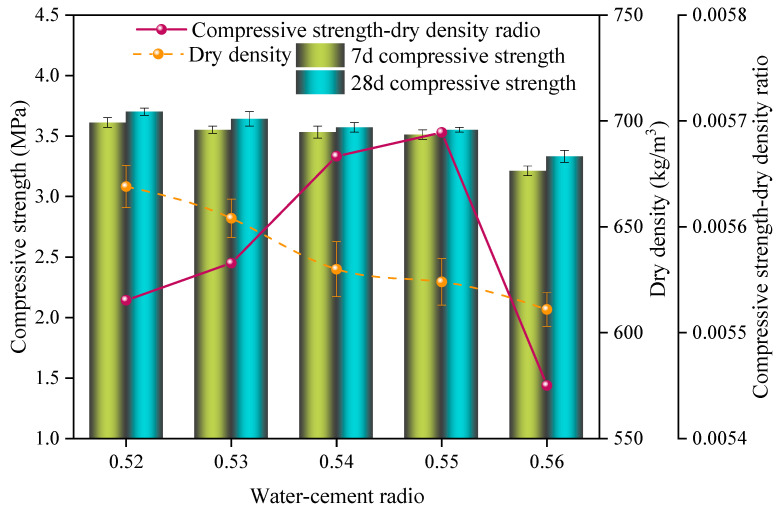
Compressive strength, dry density, and compressive strength–dry density ratio of the SAC-NAAC samples with different water–cement ratios.

Figure 2 illustrates the impact of different water–cement ratios on the properties of the SAC-NAAC samples. The 7-day compressive strength, 28-day compressive strength, and dry density of the samples declined as the water–cement ratio increased from 0.52 to 0.56.

However, the specific strength initially increased and later decreased with increasing the water–cement ratio. The highest peak specific strength was recorded at 5.68 × 10^−3^ when the water–content ratio was 0.55. The compressive strength and dry density of the SAC-NAAC sample were 3.55 MPa and 624 kg/m^3^, respectively. An increase in the water–cement ratio led to surplus water, which contributed to void formation, thereby widening the porosity of the SAC-NAAC and diminishing the strength of the material [15].

Additionally, a higher water–cement ratio reduced the viscosity of the slurry, causing the bubbles produced by the reaction of aluminum powder to coalesce into larger bubbles. This phenomenon adversely affected the strength development of the SAC-NAAC [6]. Therefore, a water–cement ratio of 0.55 was deemed optimal and selected for subsequent experiments.

### 3.2. Effect of NaOH Content on the Properties of Sulfoaluminate-Cement-Based Nonautoclaved Aerated Concrete

**Figure 3:**
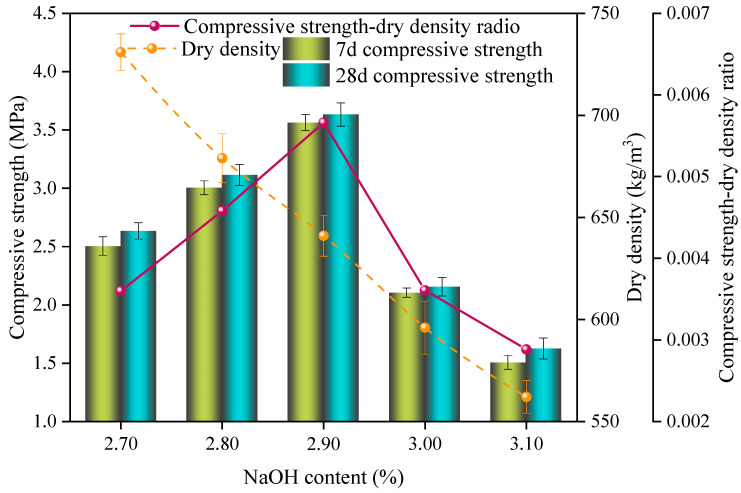
Compressive strength, dry density, and compressive strength–dry density ratio of the SAC-NAAC samples with different NaOH contents.

The influence of the NaOH content on the properties of the SAC-NAAC samples is illustrated in Figure 3. The compressive strength and specific strength of these samples initially increased and then decreased with increasing the NaOH content, whereas the dry density consistently decreased with increasing the NaOH content. At a NaOH concentration of 2.90%, the specific strength peaked at 5.66 × 10^−3^. Correspondingly, the compressive strength and dry density of the SAC-NAAC samples were recorded at 3.63 MPa and 624 kg/m^3^, respectively.

NaOH is known to facilitate the hydration of sulfoaluminate cement and create an alkaline environment conducive to the aeration process of aluminum powder [16]. When the NaOH content was less than 2.90%, the acceleration of the hydration process occurred; however, the alkalinity was insufficient for optimal aeration, leading to an inadequate aeration time for the aluminum powder and consequently smaller bubble volumes, resulting in a higher dry density of the SAC-NAAC. Moreover, continued foaming post hardening of the slurry led to air hole ruptures, negatively impacting the strength development of the SAC-NAAC samples. On the contrary, when the NaOH content exceeded 2.90%, adequate alkalinity enhanced the aeration of the aluminum powder, which increased the bubble volume while lowering the dry density.

Although an appropriate concentration of alkali can promote SAC hydration, excessive alkalinity can detrimentally affect the strength development of SAC, which may lead to cracking [17]. Thus, the optimal alkali content of the SAC-NAAC samples was 2.9%.

### 3.3. Effect of Admixture on the Properties of Sulfoaluminate-Cement-Based Nonautoclaved Aerated Concrete

**Figure 4:**
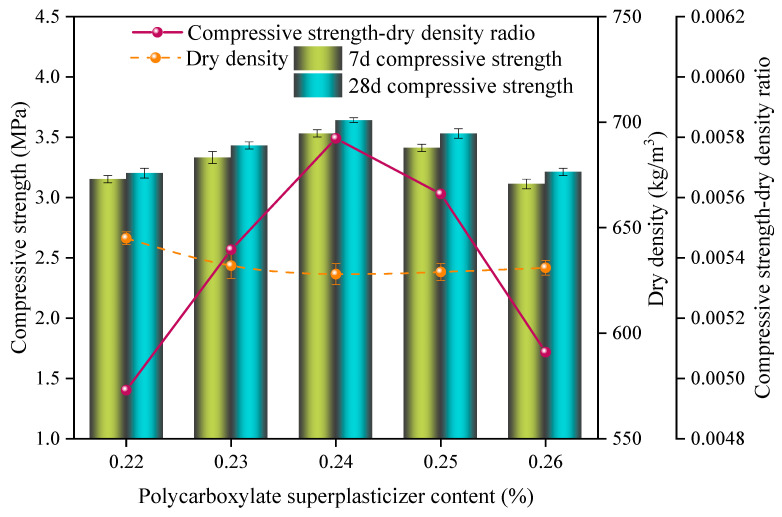
Compressive strength, dry density, and compressive strength–dry density ratio of the SAC-NAAC samples with different polycarboxylate superplasticizer contents.

The impact of different polycarboxylate superplasticizer contents on the properties of the SAC-NAAC samples is depicted in Figure 4. An initial decrease followed by an increase in the dry density was observed with the elevation of the polycarboxylate superplasticizer content, whereas the compressive strength and specific strength first increased but later declined as the content increased from 0.22% to 0.26%. The peak specific strength, 5.80 × 10^−3^, was achieved at a polycarboxylate superplasticizer content of 0.24%, with corresponding dry density and compressive strength values of 628 kg/m^3^ and 3.64 MPa, respectively.

An increase in the polycarboxylate superplasticizer content enhanced the fluidity of the slurry and reduced the resistance to aeration, leading to larger foam volumes as well as a lower dry density of the NAAC samples [18]. However, the initial setting time of the slurry remained largely unchanged; a lower aeration resistance implied a quicker aeration rate. If the aeration time of the aluminum powder was shorter than the initial setting time of the slurry, the slurry would collapse, reducing the volume of the SAC-NAAC and increasing its dry density. Therefore, the optimal polycarboxylate superplasticizer content was determined to be 0.24%.

**Figure 5:**
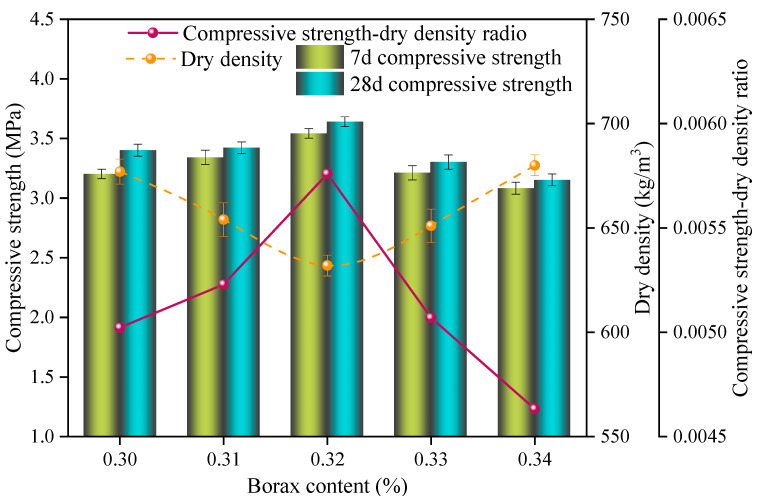
Compressive strength, dry density, and compressive strength–dry density ratio of the SAC-NAAC samples with different borax contents.

Similarly, the effect of different borax contents on the SAC-NAAC samples is shown in Figure 5. Within the range of 0.30–0.34% borax content, the dry density initially decreased and then increased with increasing the borax content, and the compressive strength and specific strength exhibited a converse trend. The highest specific strength, 5.76 × 10^−3^, was observed at a borax content of 0.32%, with a corresponding dry density and compressive strength of 632 kg/m^3^ and 3.64 MPa, respectively. The ionized B_4_O_7_^2−^ from borax interacted with Ca^2+^ in the SAC slurry to produce calcium borate, forming a protective film around the cement particles, thereby delaying the setting time and providing sufficient time for the gassing of the aluminum powder [19]. When the borax content was below 0.32%, ongoing aeration post coagulation led to a ruptured pore structure connectivity, resulting in an increased dry density and reduced strength of the SAC-NAAC samples. On the contrary, when the borax content exceeded 0.32%, the setting time of the slurry was longer than the aeration time of the aluminum powder. This caused smaller bubbles to float up and coalesce into larger, interconnected bubbles and even result in mold collapse, which eventually increased the dry density and decreased the strength. Therefore, a borax content of 0.32% was considered ideal for synchronizing the aeration time with the setting time of the slurry.

### 3.4. Effect of Different Aluminum Powder Contents on the Performance of Sulfoaluminate-Cement-Based Nonautoclaved Aerated Concrete

**Figure 6:**
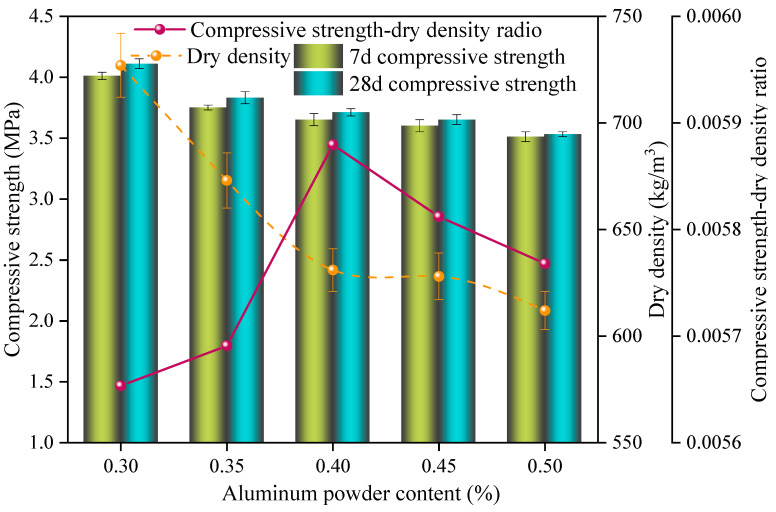
Compressive strength, dry density, and compressive strength–dry density ratio of the SAC-NAAC samples with different aluminum powder contents.

The effect of different aluminum powder contents on the properties of the SAC-NAAC samples is displayed in Figure 6. As the aluminum powder content increased, the compressive strength and dry density of the samples decreased, and the specific strength initially increased before declining. The highest specific strength, recorded at 5.88 × 10^−3^, was achieved with an aluminum powder content of 0.40%. At this concentration, the compressive strength was measured at 3.71 MPa and the dry density was 620 kg/m^3^. Consequently, an aluminum powder content of 0.4% was identified as optimal for producing B06 A3.5 grade nonautoclaved aerated concrete.

**Figure 7:**
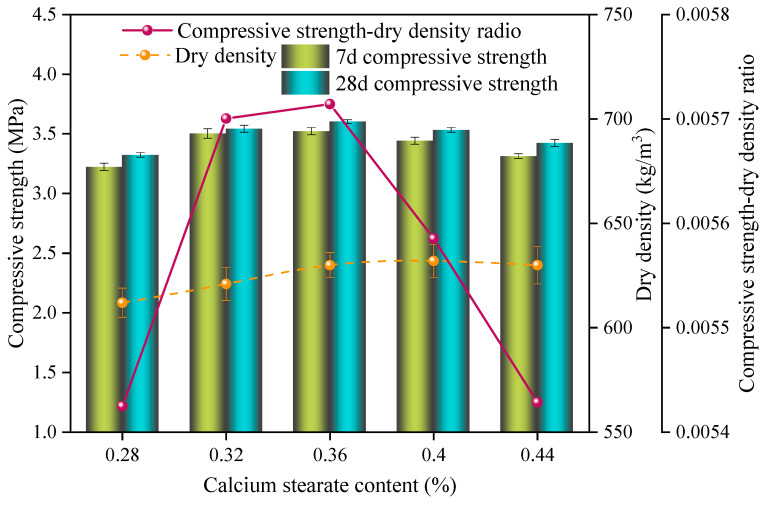
Compressive strength, dry density, and compressive strength–dry density ratio of the SAC-NAAC samples with different calcium stearate contents.

The impact of different calcium stearate contents on the SAC-NAAC samples is depicted in Figure 7. The dry density of the samples initially increased and then stabilized, and the compressive strength and specific strength first intensified and then declined with increasing the calcium stearate content. The peak specific strength of 5.72 × 10^−3^ was observed at a calcium stearate content of 0.36%, where the dry density of the NAAC was 630 kg/m^3^ and the compressive strength was 3.6 MPa. By incorporating a suitable amount of foam stabilizer, calcium stearate can enhance the pore structure of AAC [20].

When the calcium stearate content was below 3.6%, the pore structure of the SAC-NAAC samples became increasingly uniform with the calcium stearate content. However, when the calcium stearate content was above 3.6%, an increase in the calcium stearate content led to a heightened slurry viscosity and greater resistance to aeration, resulting in the initial setting time of the slurry being insufficient to support the complete aeration process of the aluminum powder, thereby resulting in cracking. Calcium stearate acts as a foam stabilizer owing to its low density and hydrophobic properties [21]. Nonetheless, excessive calcium stearate could accumulate on the paste surface during mixing or even clump together, forming weak areas.

This aggregation undermined the effect of foam stabilization, adversely affecting the mechanical properties of the autoclaved aerated concrete [22]. Therefore, the optimal calcium stearate content was established at 0.36%.

**Figure 8:**
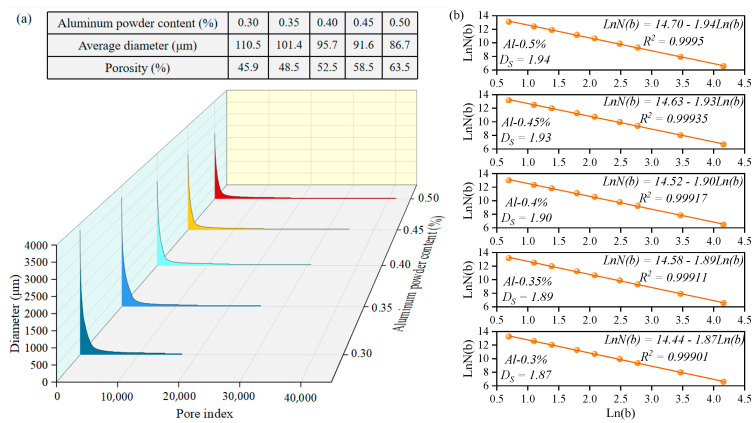
(**a**) Porosity and average diameter and (**b**) fractal dimension of the SAC-NAAC samples with different aluminum powder contents.

Figure 8a displays the porosity and average pore diameter of the SAC-NAAC samples with different aluminum powder contents. An increase in the aluminum powder content resulted in a heightened porosity and reduced average pore diameter. Specifically, the porosities were 45.9%, 48.5%, 52.5%, 58.5%, and 63.5% for aluminum powder ranges of 0.30–0.50%, and their average diameters were 110.5, 101.4, 95.7, 91.6, and 86.7 µm, respectively. As the aluminum powder content increased, the number of pores in the SAC-NAAC samples escalated, whereas the pore diameter decreased. The respective pore numbers for the different aluminum powder contents were 17,467, 25,859, 30,879, 34,675, and 41,669. Additionally, Figure 8b reveals the fractal dimension of the SAC-NAAC samples with different aluminum powder contents, indicating a reduction in the fractal dimension of the pore distribution from 1.94 to 1.87 with an increase in the aluminum powder content. These findings indicate that the pore distribution became more homogeneous as the aluminum powder content increased.

**Figure 9:**
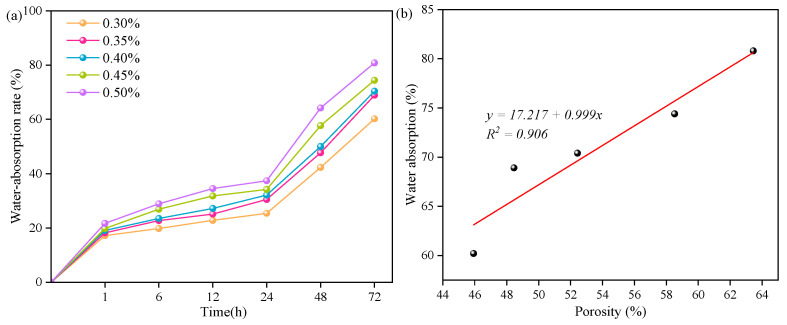
(**a**) Water absorption and (**b**) water absorption–porosity relation of the SAC-NAAC samples with different aluminum powder contents.

Figure 9a illustrates the influence of different aluminum powder contents on the water absorption of the SAC-NAAC samples. The water absorption mass of the samples at various absorption times escalated with an increase in the aluminum powder content. The 72 h water absorption rates for the samples with different aluminum powder contents were 60.2%, 68.9%, 70.4%, 74.4%, and 80.8%. Furthermore, water absorption in AAC is primarily influenced by the number and structure of pores (uniformity, physical state, etc.). A higher porosity correlates with thinner pore walls and a reduced pore confinement performance [23]. Figure 9b depicts the relationship between the water absorption and porosity in the SAC-NAAC samples with different aluminum powder contents. A notable linear correlation (*y* = 17.217 + 0.999x, and *R*^2^ = 0.906) was observed between the water absorption and porosity for aluminum powder contents ranging from 0.30% to 0.50%. Thus, the water absorption of the samples increased proportionally with their porosity.

**Figure 10:**
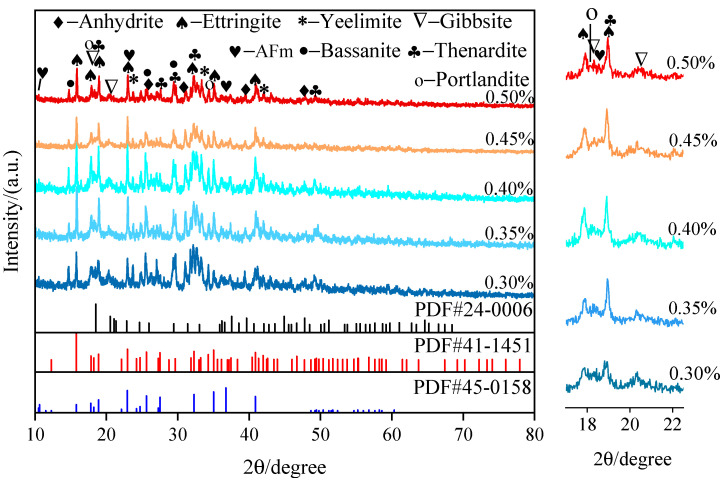
XRD patterns of the SAC-NAAC samples with different aluminum powder contents.

The XRD patterns of the SAC-NAAC samples with varied aluminum powder contents are displayed in Figure 10. The mineral composition of the samples predominantly comprised ettringite (AFt, PDF#41-1451), yeelimite (PDF#33-0256), gibbsite (AH_3_, PDF#24-0006), monosulfide aluminum sulfate (AFm, PDF#45-0158), thenardite (PDF#37-1465), portlandite (PDF#04-0733), and gypsum (PDF#43-0606 and PDF#41-0224). The early hydration reaction of the SAC primarily produced AFt and AH_3_, as expressed in Equations (1) and (2).

(1) $$\begin{array}{ll} 3CaO\cdot Al_{2}O_{3}\cdot & H_{2}O+3CaSO_{4}\cdot {2H}_{2}O+25H_{2}O\\ & \;=3CaO\cdot Al_{2}O_{3}\cdot 3CaSO_{4}\cdot 32H_{2}O, \end{array}$$

(2) $$\begin{array}{c} CaO+4Al_{2}O_{3}+SO_{3}+2CaSO_{4}\cdot H_{2}O+39H_{2}O=3CaO\cdot Al_{2}O_{3}\cdot 3CaSO_{4}\cdot \\ 32H_{2}O+3\left(Al_{2}O_{3}\cdot 3H_{2}O\right), \end{array}$$

AFt is the principal hydration product, greatly contributing to the early strength of SAC [24]. The content of AFt in the hydration product increased, followed by a decrease with increasing the aluminum powder content. The addition of NaOH altered the progression of the original hydration reaction, with the high-alkalinity environment impacting AFt formation [25]. After the addition of NaOH, SAC reacted with NaOH as follows (Equations (3) and (4)):

(3) $$CaSO_{4}+2NaOH=Na_{2}SO_{4}+Ca(OH{)}_{2},$$

(4) $$\begin{array}{c} 3CaO\cdot Al_{2}O_{3}\cdot 3CaSO_{4}\cdot 32H_{2}O+2\left(3CaO\cdot Al_{2}O_{3}\cdot H_{2}O\right)+2H_{2}O=3(3CaO\cdot \\ Al_{2}O_{3}\cdot CaSO_{4}\cdot 12H_{2}O), \end{array}$$

Anhydrite, which is highly active in a high-alkalinity environment, reacted rapidly with NaOH to form thenardite and portlandite. Yeelimite reacted with excess NaOH to form AH_3_, thenardite, and portlandite. The precipitation of thenardite reduced the alkalinity of the concrete to a certain extent and promoted the generation of AFm, which manifested in a small number of characteristic peaks of AFm [26]. Moreover, the low-density thenardite could transform into mirabilite (Na_2_SO_4_·H_2_O), resulting in an increased concrete volume and porosity, which directly affected the mechanical properties of the SAC-NAAC samples [27]. AH_3_ was observed as a weak characteristic peak due to its occurrence in a microcrystalline form [28]. Therefore, the inclusion of aluminum powder and calcium stearate merely altered the generation of different mineral components without changing the mineral composition of the SAC [22].

**Figure 11:**
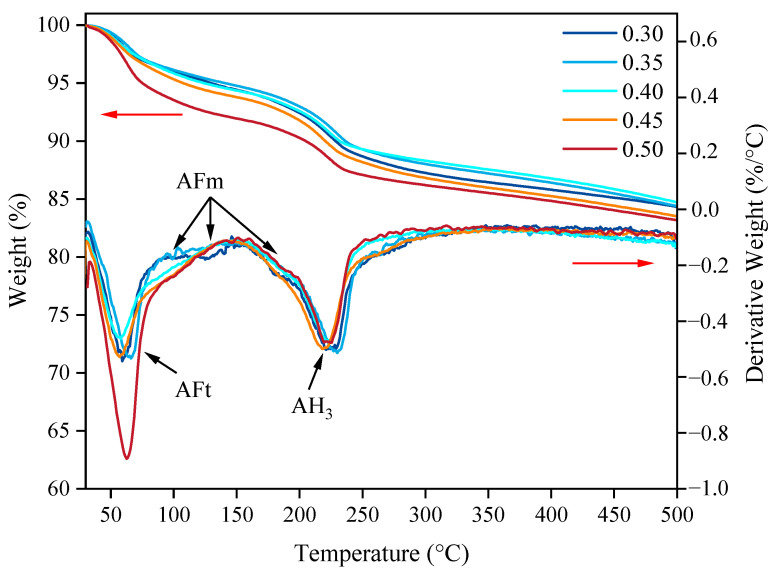
TG–DTG curves of the SAC-NAAC samples with different aluminum powder contents.

The thermogravimetric (TG) and derivative thermogravimetric (DTG) curves of the SAC-NAAC samples with different aluminum powder contents are illustrated in Figure 11. The primary weight loss components in these samples were AFt, AFm, and AH_3_. The dehydration temperature range for AFt was below 150 °C, for AFm, it spanned from 150 °C to 200 °C, and for AH_3_, it ranged from 200 °C to 300 °C. Moreover, the weight loss rates for the samples with aluminum powder contents of 0.3%–0.5% were 15.69%, 15.62%, 15.23%, 16.49%, and 16.83%. Therefore, the dehydration temperatures of the hydration products of the SAC-NAAC samples were consistent for the different aluminum powder contents, aligning with the XRD findings. The AH_3_ contents in the SAC-NAAC samples were nearly identical, regardless of the aluminum powder content, demonstrating that the aluminum powder quantity did not influence AH_3_ formation.

**Figure 12:**
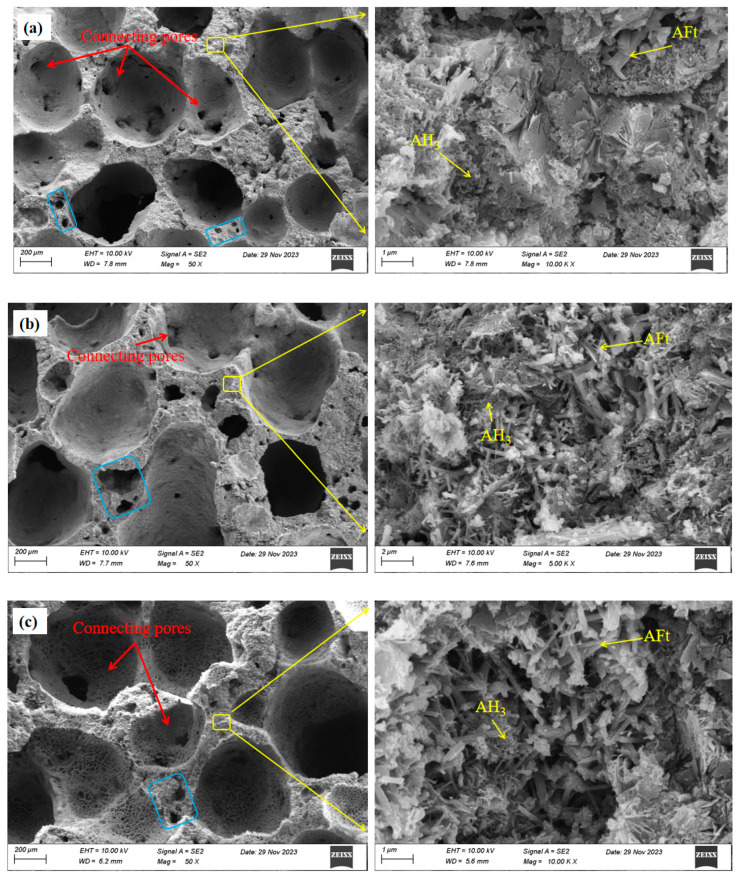

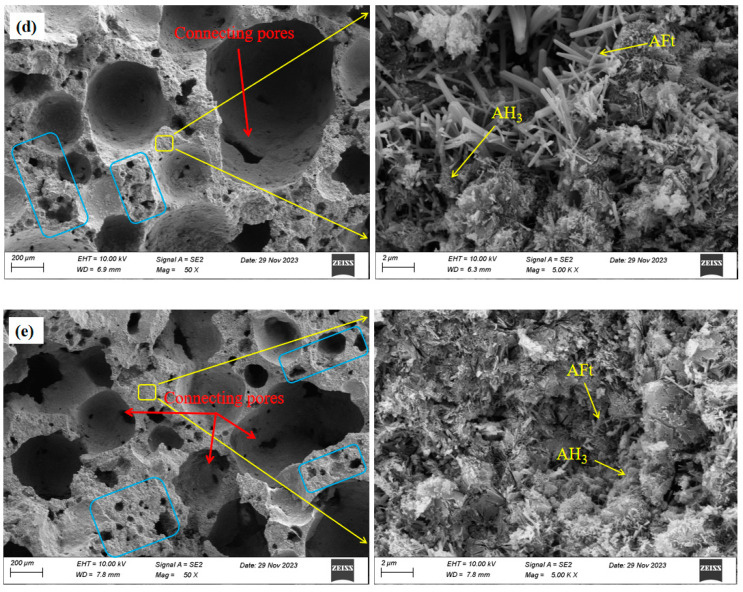
SEM images of the SAC-NAAC samples with different aluminum powder contents. (**a**) 0.30%; (**b**) 0.35%; (**c**) 0.40%; (**d**) 0.45%; (**e**) 0.50%.

Figure 12 presents the SEM images of the pore structure and crystal morphology of the SAC-NAAC samples with different aluminum contents. An increase in the aluminum powder content led to a gradual decrease in the pore diameter and pore wall thickness, while the number and size of connecting holes in the pore walls escalated. As depicted in Figure 12a–d, the pore structure exhibited connecting pores when the aluminum powder content ranged between 0.30% and 0.50%. However, the pore wall thickness of the SAC-NAAC samples diminished, and the number of connecting pores increased with the increase in the aluminum powder content [29]. Moreover, the number of small holes in the pore walls increased with the aluminum powder content, which was attributable to the evaporation of free water in tiny bubbles formed during slurry mixing and the secondary reaction of excess aluminum powder. This accounted for the rapid rise in the small pore numbers with the increase in the aluminum powder content in the previous pore size distribution section. The augmented number of small holes and connecting pores in the pore walls was correlated with the decreased mechanical properties of the SAC-NAAC samples [30,31,32]. The relationship between the porosity and compressive strength of the samples conformed to the exponential model (Equation (5)) proposed by Ryshkevitch [33,34].

(5) $$\sigma_{c}=\sigma_{0}\exp(-np),$$

where *σ*_c_ denotes the compressive strength of the concrete with the porosity (*p*), *σ*_0_ indicates the compressive strength at zero porosity, *p* refers to the porosity (volume of voids expressed as a fraction of the total concrete volume), and *n* denotes a coefficient. Therefore, the porosity of the SAC-NAAC samples increased and the compressive strength decreased with the increase in the aluminum powder content. This was consistent with the findings on the mechanical properties and pore size distribution of the SAC-NAAC samples with different aluminum powder contents.

Hexagonal prismatic crystals of AFt are the primary hydration products [24]. Despite the expectation of a substantial AH_3_ presence in the hydration product, not all of it was detectable in the XRD pattern, as AH_3_ frequently occurs in microcrystalline or amorphous phases [28]. As observed in Figure 12d, well-crystallized AH_3_ adhered around the AFt skeleton, creating a dense structure. This formation is advantageous for filling the pores in the porous material and enhancing the strength of SAC-NAAC [35].

The XRD results demonstrate that the strength of the SAC-NAAC originated from the dense AFt–AH_3_ structure. As depicted in Figure 12a, the aluminum powder content was at 0.30%, and the overall structure of the SAC-NAAC appeared to be relatively dense with robust AFt crystal patterns. As the aluminum powder content increased, the structure became progressively looser and the AFt crystal pattern slimmed down. However, the acceleration of the hydration process in the highly alkaline environment resulted in unstable AFt formation, deteriorating the mechanical properties of the SAC-NAAC [36]. An analysis of the XRD results further revealed that the quantity of AFt produced initially increased and then decreased with the increase in the aluminum powder content, thereby verifying the observed trend in the specific strength variations from the aforementioned mechanical experiments.

## 4. Conclusions

(1) Compared to conventional autoclaved aerated concrete (AAC) and the existing nonautoclaved aerated concrete (NAAC), a sulfoaluminate-cement-based nonautoclaved aerated concrete (SAC-NAAC) was prepared by reducing the high-temperature autoclave process. It not only reduced the consumption of energy and filled the vacancy of preparing NAAC at 20 ± 2 °C, but the shorter setting time of the paste also greatly shortened the production cycle of the SAC-NAAC, laying the foundation for its mass production.
(2) The dry density and compressive strength of the SAC-NAAC gradually decreased with the increase in the W/C and aluminum powder content. Conversely, as the NaOH content increased, the compressive strength first increased and then decreased, whereas the dry density decreased linearly. This phenomenon was attributed to the heightened alkalinity accelerating the aeration speed and the resulting mismatch between the slurry setting and aeration periods. Optimal physical properties of the slurry were achieved by adding additives. At a W/C ratio of 0.55 and a specific content ratio of polycarboxylate superplasticizer–borax–calcium stearate–sodium hydroxide of 0.24%:0.32%:0.36%:2.90%, the setting time of the slurry aligned with the aeration time of the aluminum powder, resulting in the optimal foaming state.
(3) An increase in the aluminum powder content led to an increase in the number of pores in the SAC-NAAC, a reduction in the average pore diameter, and the formation of thinner pore walls with small pores. This process resulted in an increased number of connected pores, elevating the porosity and bulk density of the SAC-NAAC. Additionally, the water absorption intensified with the increase in the aluminum powder content due to a greater number of connected pores in the low-density SAC-NAAC, which increased the porosity. Consequently, the correlation between the water absorption and porosity of the SAC-NAAC could be expressed as a linear function.
(4) AFt generated via SAC hydration provided early strength to the SAC-NAAC, and only a limited amount of AH_3_ was formed via SAC hydration. The introduction of NaOH altered the hydration process of yeelimite, and the high-alkalinity environment facilitated the formation of AH_3_ and the conversion of a portion of AFt into AFm. AH_3_ adhered to the surface of prismatic AFt in a microcrystalline gel state, filling the AFt skeleton and creating a dense structure, which further enhanced the mechanical properties of the SAC-NAAC.

## Figures and Tables

**Table 1:** Main constituents of sulfoaluminate cement.

SiO_2_	Al_2_O_3_	Fe_2_O_3_	CaO	MgO	TiO_2_	SO_3_
<10.5	>34	1.5–3.5	41.5–43.5	<3.5	1.0–2.0	7.5–9.5

## Data Availability

Data are contained within the article.
